# Diffuse idiopathic skeletal hyperostosis as an overlooked cause of dysphagia: a case report

**DOI:** 10.1186/1752-1947-2-287

**Published:** 2008-08-27

**Authors:** Seema Srivastava, Natalia Ciapryna, Iñaki Bovill

**Affiliations:** 1 Department of Elderly Care, Chelsea and Westminster Foundation Hospital, Fulham Road, London, SW10 9NH, UK

## Abstract

**Introduction:**

Dysphagia is a common presentation in older people. Diffuse idiopathic skeletal hyperostosis affecting the cervical spine is an uncommon cause of dysphagia and may be overlooked.

**Case presentation:**

We present the case of an 88-year-old man with dysphagia and weight loss. Initial investigation with upper gastrointestinal endoscopy was inconclusive. A diagnosis of diffuse idiopathic skeletal hyperostosis as a cause for dysphagia was eventually made using video fluoroscopy. This showed a bony prominence impeding swallow at the level of C3. The patient was unfit for surgical management so a percutaneous endoscopic gastrostomy tube was inserted for feeding.

**Conclusion:**

The diagnosis of diffuse idiopathic skeletal hyperostosis involving the cervical spine often goes unrecognised as a cause of dysphagia despite its prevalence in the elderly population. Diagnosis is made using cervical radiographs, barium swallow and computed tomography. There is a risk of perforation with endoscopy in patients who have cervical diffuse idiopathic skeletal hyperostosis. Conservative management includes non-steroidal anti-inflammatory medications and a modified diet. Surgery may be considered in certain patients where conservative management fails.

## Introduction

Diffuse idiopathic hyperostosis was first described in 1950 by Forestier and Rotes-Querol [1]. It is characterised by excessive ligamentous calcification and ossification at spinal and extraspinal locations. When the cervical spine is involved large osteophytes may form, causing symptoms of dysphagia. We describe the case of an 88-year-old man with dysphagia and weight loss secondary to diffuse idiopathic skeletal hyperostosis (DISH).

## Case presentation

An 88-year-old man presented with a 6-month history of dysphagia for solid foods and significant weight loss. He denied any symptoms of odynophagia. He denied any hoarseness of the voice, neck pain or breathlessness. There was no change in bowel habit or blood in the stools. His calorific intake was solely dependent on protein supplement drinks. His previous medical history was of type 2 diabetes, hypercholesterolaemia, hypertension, atrial fibrillation and glaucoma.

**Figure 1:**
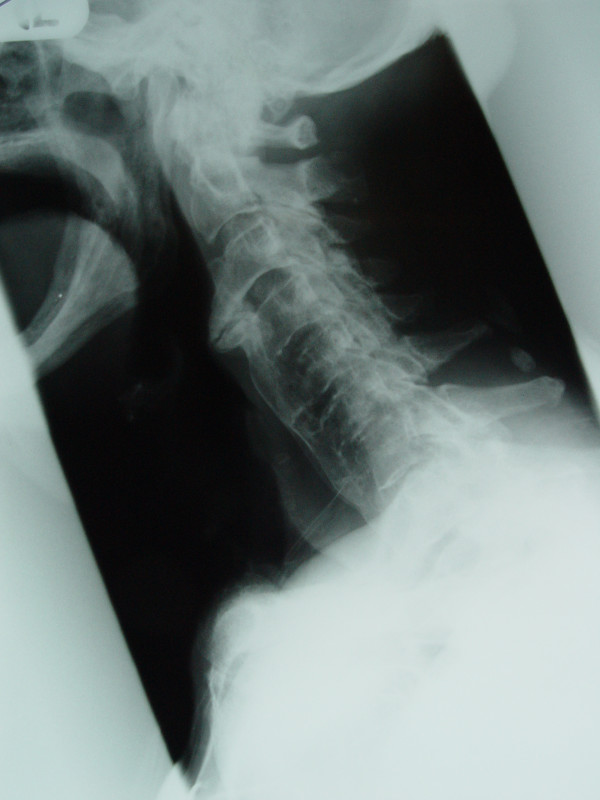
Lateral radiograph of the cervical spine showing anterior osteophyte formation most marked at the C3/C4 vertebrae and calcification of the anterior longitudinal ligaments.

On examination he was cachetic and pale. His weight was 54 kg. The rest of his physical examination was unremarkable. His full blood count showed a normocytic anaemia (10.3 g/dl) with a normal ferritin level. Liver function was normal apart from an albumin of 28 g/l. Erythrocyte sedimentation rate and thyroid stimulating hormone were normal. An endoscopy was performed to exclude an intrinsic cause for the patient's symptoms. This showed chronic atrophic gastritis but no cause for the dysphagia. Video fluoroscopy was performed which showed a bony prominence impeding swallow at the level of C3. A lateral cervical spine radiograph showed anterior osteophyte formation, most marked at the C3/C4 vertebrae and consistent with DISH (Figure 1).

He was commenced on nasogastric feeding, as there was evidence of aspiration on video fluoroscopy. He was referred to the spinal surgeons but they did not feel surgery was appropriate due to the patient's frail condition and comorbidities. A percutaneous endoscopic gastrostomy tube was placed 3 weeks later. The patient died 6 weeks after admission, from complications secondary to an unrelated septic arthritis of the shoulder.

## Discussion

DISH is a common but overlooked condition seen in the elderly. It is characterised by new bone formation into axial and peripheral enthesial regions. The prevalence of DISH has been reported to be 10% in patients over the age of 70 (see [2]). The aetiology of DISH has not been defined but there are associations with diabetes, obesity [3], hypercholesterolaemia and gout. DISH most commonly affects the thoracic spine although cervical involvement is found in 76% of those affected [4]. Dysphagia related to DISH affecting the cervical spine has a reported prevalence of 28% [5]. Dysphagia caused by DISH may be due to several factors: direct mechanical compression of the oesophagus by large anterior osteophytes; smaller osteophytes located at sites of oesophageal fixation such as at the level of the cricoid cartilage; inflammation of the peri-oesophageal soft tissue in contact with overlying osteophytes; or oesophageal spasm caused by painful osteophytes [6].

The diagnosis of DISH is radiological. Plain radiographs of the cervical spine typically show flowing calcification and ossification along the anterior surface of at least four contiguous vertebrae. Large anterior osteophytes are commonly found between C4 and C7 [7]. Computed tomography is another useful imaging modality in the diagnosis of DISH as the size and shape of the osteophytes are shown in relation to the oesophagus and other important structures. Barium swallow or video fluoroscopy will confirm oesophageal compression and obstruction in relation to large anterior osteophytes. Endoscopy in these patients carries a risk of perforation but may be necessary to exclude other intrinsic causes of dysphagia such as oesophageal strictures, oesophagitis, oesophageal webs, motility disorders, tumours and candidiasis [8]. Other clinical manifestations associated with cervical DISH are hoarseness, stridor, aspiration pneumonia, myelopathy, thoracic outlet syndrome and sleep apnoea [7]. Treatment is divided between conservative and surgical. Conservative management includes modification of diet, non-steroidal inflammatory medications, corticosteroids and muscle relaxants [9,10]. In severe cases surgical management may be the only option and involves osteophytectomy. The surgical approach may be anterolateral, posterolateral or transpharyngeal when C2 to C4 vertebrae are involved. Complications include laryngeal nerve damage, stroke, Horner's syndrome and cervical instability [11].

## Conclusion

Dysphagia is a common presentation seen in older people. The diagnosis of DISH involving the cervical spine often goes unrecognised as a cause of dysphagia despite its prevalence in the elderly population. Diagnosis is established with plain cervical radiographs and barium swallow especially when endoscopy has excluded an intrinsic cause for dysphagia.

## Abbreviations

DISH: Diffuse idiopathic skeletal hyperostosis.

## Competing interests

The authors declare that they have no competing interests.

## Authors' contributions

The authors were involved in the writing of the manuscript or patient clinical care. All authors read and approved the final manuscript.

## Consent

Written informed consent could not be obtained in this case since the patient's next-of-kin were untraceable. We believe this case report contains a worthwhile clinical lesson which could not be as effectively made in any other way. We expect the patient's next-of-kin not to object to the publication since every effort has been made so the patient remains anonymous.
